# The Springer Model for Lifetime Prediction of Wind Turbine Blade Leading Edge Protection Systems: A Review and Sensitivity Study

**DOI:** 10.3390/ma15031170

**Published:** 2022-02-03

**Authors:** Nick Hoksbergen, Remko Akkerman, Ismet Baran

**Affiliations:** Faculty of Engineering Technology, University of Twente, Drienerlolaan 5, 7522 NB Enschede, The Netherlands; r.akkerman@utwente.nl (R.A.); i.baran@utwente.nl (I.B.)

**Keywords:** Springer, lifetime prediction, wind turbine blade, droplet impact, LEP

## Abstract

The wind energy sector is growing rapidly. Wind turbines are increasing in size, leading to higher tip velocities. The leading edges of the blades interact with rain droplets, causing erosion damage over time. In order to mitigate the erosion, coating materials are required to protect the blades. To predict the fatigue lifetime of coated substrates, the Springer model is often used. The current work summarizes the research performed using this model in the wind energy sector and studies the sensitivity of the model to its input parameters. It is shown that the Springer model highly depends on the Poisson ratio, the strength values of the coating and the empirically fitted $a_{2}$ constant. The assumptions made in the Springer model are not physically representative, and we reasoned that more modern methods are required to accurately predict coating lifetimes. The proposed framework is split into three parts—(1) a contact pressure model, (2) a coating stress model and (3) a fatigue strength model—which overall is sufficient to capture the underlying physics during rain erosion of wind turbine blades. Possible improvements to each of the individual aspects of the framework are proposed.

## 1. Introduction

**Figure 1:**
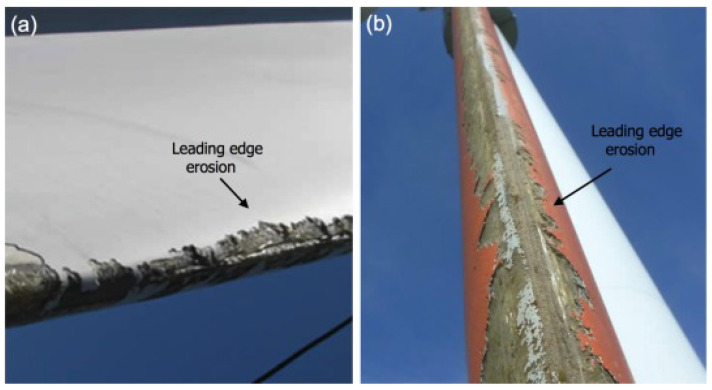
Leading edge erosion damage on two blades (**a**) and (**b**) (reprinted from [11], with permission from Elsevier).

Global climate accords demand lower CO_2_ emissions. Production of renewable energy using wind turbines shows high potential towards achieving these goals. In order to produce more energy from wind turbine blades, they are being made larger and currently exceed 200 m in diameter. These large wind turbines are prone to different types of damage [1,2]. The tip velocities of over 100 ms${}^{-1}$ cause interactions of the blades with rain droplets and other airborne particles, leading to erosion damage over time [3]. The erosion damage, as shown in Figure 1, leads to a decrease in aerodynamic efficiency and hence a reduction in annual energy production (AEP) [4,5,6]. Regular costly maintenance has to occur to maintain structural integrity and aerodynamic efficiency of the blades [7]. Lately, leading edge protection (LEP) systems have been developed that consist of erosion resistant materials to elongate maintenance intervals [8] while limiting the AEP loss [9]. A commonly used model to estimate the lifetime of such LEP systems was developed by Springer [10].

The main challenge in the field is to accurately predict lifetimes for coated wind turbine blades based on easily obtainable material properties. Regular, long duration, expensive experimental rain erosion testing is performed to obtain lifetime curves. These VN curves can be fitted to material parameters using the Springer model to allow prediction of lifetime performance outside the tested velocities. This method, however, does not allow accurate prediction of lifetimes for different materials or impact conditions.

Since the Springer model is used as the basis for most current work on lifetime prediction for leading edge protection systems for wind turbine blades, it is revisited in the current paper. An objective of this paper is to critically assess the sensitivity of the Springer model to its input parameters for typical coating materials, which has not been done before. In addition, improvements to the Springer model based on modern methods will be proposed, allowing for a clear course for future research to enable more accurate lifetime prediction.

**Figure 2:**
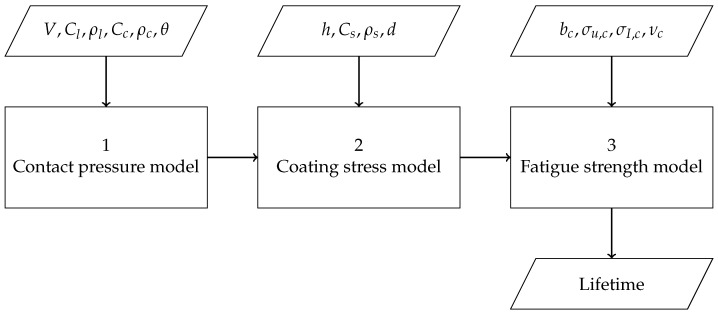
Overview of the Springer model.

The Springer model, first mentioned in 1972 [12], is still the most used model for lifetime prediction of coating systems for wind turbine blades today. For the purpose of this paper, it is split into three parts, as shown in Figure 2. The first part determines the pressure that the liquid droplet exerts on the LEP system. The second part determines the stress in the LEP system based on the contact pressure. The third part uses the computed stress to calculate the LEP system lifetime based on its fatigue properties. The model considers a liquid column impacting a (coated) elastic substrate. It takes into account the stress in one dimension, through thickness. Furthermore, the lifetime calculation is based on the Wöhler fatigue data of the impacted material. The implementation of the individual parts will be further discussed in this paper and improvements based on more modern methods will be proposed.

The Springer model is used for several type of blade, including steam turbine blades [13,14], aircraft propellers and wings and wind turbine blades. Especially in the latter category, current research is focused towards new types of coatings that enhance lifetime.

The Springer model was used as the basis for several coating lifetime studies. It was used to predict both the incubation periods and the erosion rates for typical gelcoat materials, and showed good correlations with rain erosion experiments and field data [15]. In combination with the Palmgren–Miner rule [16], an engineering approach to track cumulative surface fatigue damage was developed based on the maximum Rayleigh wave stress. It was argued that in order to extend lifetime, a low contact pressure and a large safe area for fatigue damage are required. In combination with a dispersed jet based test setup, the developed model was used to predict and verify erosion damage on polybutylene terephthalate (PBT) polymers [17], along with several ferrous and non-ferrous metals [18].

In order to enable site-specific analysis for rain erosion performance and to generate rain droplet distribution fields, meteorological data are required [19]. The meteorological data are used to predict erosion behavior based on the Springer model. It has been shown that offshore locations have more severe rain conditions than inland locations in several areas, such as Denmark [20,21], the British Isles [22,23,24] and Netherlands [25]. In the USA, inland conditions vary heavily, as measured by a radar [26], yet they have a significant effect on leading edge lifetime. The ability to measure in situ the presence and intensity of rain and when appropriate scale down a wind turbine’s rotational velocity has been shown to decrease erosion damage [27], but this also results in lower power output by the turbine.

Machine learning based methods were applied to investigate the effect of erosion on the AEP of wind turbines [28,29]. The erosion of the blades was assumed to develop according to the Springer model. The results showed that eroded blades have a lower aerodynamic efficiency causing a lower AEP, which corresponded well with measured field data. Machine learning methods [30] could be utilized in order to predict the mechanical performance of the LEP system’s components.

Environmental effects, such as other airborne particles, temperature changes, UV-degradation, droplet size distributions and local wind speeds, were coupled with the Springer model to enable site-specific analysis of rain erosion damage [25,31,32]. In some cases the modeled erosion behavior corresponded well with field data, and in other cases it did not.

Repeatable and reproducible rain erosion test (RET) methods have been developed throughout the years. Two main categories are available: jet based methods and whirling arm based methods [33]. The latter is the most accepted and studied method [34], which led to it being recommended practice (DNVGL-RP-0171) [35]. Lately, a “single point impact fatigue test (SPIFT)” [36] method based on rubber ball-impact was developed and used to study polyurethane LEP systems [37]. To quantify the amount of erosion damage, mass loss or volume loss is generally computed. This can be based on surface analysis or weighing of the damaged LEP specimens [38]. The velocity where the damage initiation is observed is plotted against the number of impacts or the exposure time and related to the Springer model predictions using the waterhammer pressure to obtain a VN curve.

The basic Springer model was further investigated for viscoelastic coating materials, and it was found that the required input data are very sensitive to the applied strain rate [39,40] by testing the acoustic properties at several frequencies using time of flight measurements of piezo-electric induced waves. As the high-strain rate strength properties are often hard to obtain, an approach where the strength data are fitted to rain erosion experiments was proposed [41]. In this work, the $a_{2}$ constant was fitted using linear regression and found to fit best for values between 7.28 and 11.85 for these particular materials. The ratio of $\sigma_{e,c}$ and $\sigma_{0,c}$ was changed to shift the curve.

It was shown that for particular coating systems, the interface between the coating and the substrate plays an important role in the performances of LEP systems both in terms of stress reflections and adhesion strength [42]. Poor adhesion or local manufacturing defects can lead to an early onset of damage [43].

For thermoplastic based protection systems, the damage initiation and propagation mechanisms were studied experimentally and successfully correlated with numerical simulations [44] in terms of damage type and location. More work towards lifetime prediction, however, is required. The interphase for a thermoplastic LEP that is co-bonded to a thermoset substrate was studied extensively [45,46]. A delicate balance between inter-diffusion and cure-kinetics was observed and linked to processing conditions and interphase quality [47].

A comparison of several analytical descriptions of the contact pressure and impact force with numerical models [48,49] showed a good correlation. A numerical study used a full 3D multiaxial critical plane fatigue model to estimate coating degradation [50]. An interpolation of numerically computed droplet impact pressures was used to compute coating lifetimes [11,51]. Further numerical studies showed that the contact pressure for liquid droplet impact on a rigid substrate depends on time, droplet size and distance to the center of impact [52,53]. A numerical framework for determining the lifetime of a coated wind turbine blade was proposed, although it did not consider target elasticity. An analytical description for low velocity liquid droplet impact on elastic substrates was derived based on a set of integro-differential equations. This approach includes air cushioning [54] and describes the coupling between the free surfaces of the domains [55]. This work showed that the dynamic impact pressure highly depends on the impact, material and geometric parameters of the problem.

The effects of defects and fillers in a polymeric coating on the damage mechanisms have been investigated numerically [56] and verified experimentally using several imaging techniques [57]. A micromechanical model was developed for investigation of surface erosion in segregated polyurethane coatings [58]. Air bubbles in the coating had a significant influence on leading edge lifetime due to stress concentrations around these defects [59]. The micromechanical failure mechanisms were studied using X-ray tomography based methods [60], further substantiating the effect of stress concentrations around defects or particle inclusions in the coating. Moreover, the effects of environmental and design variables were studied numerically [61]; and it was shown that droplet shape, surface wetness and damping properties of the coating have significant influences on the resulting stress fields. Advanced numerical methods such as extended isogeometric analysis (XIGA) [62] could be used to computationally study the fracture mechanics of LEP systems.

Other parameters, such as blade curvature [63] and impact velocity and angle [64], have also been studied. It was shown that all three parameters might complicate the analysis of the problem, since their behavior differed from what was assumed by the Springer model.

It is well known that liquid droplet impact causes high contact pressures and that the dynamics involved are complex. In order to develop reliable protection systems for wind turbine blades, a fundamental understanding of the involved physics is required, and this should be incorporated in the models [65,66].

## 2. The Springer Model

This section describes the governing equations in the Springer model adapted from [10,12,67]. For convenience, it is split into the equations for an uncoated substrate, followed by those for a coated substrate. The used quantities and units are summarized in Table 1.

### 2.1. Impact on an Uncoated Solid

The simplest form of the Springer model considers liquid droplets’ impact on an infinitely thick elastic substrate without a coating. In this model, the contact pressure between a liquid droplet and the elastic substrate is assumed to be equal to the (modified) waterhammer pressure given in Equation (1). This equation depends on the impact velocity (*V*), the impact angle (θ) and the densities (ρ) and acoustic velocities (*C*) of both the liquid and the solid denoted by the subscripts *l* and *s*, respectively. The density and acoustic velocity can be replaced by the acoustic impedance Z=ρC.

(1) $$\sigma_{1,s}=\frac{\cos\left(\theta \right)VZ_{l}Z_{s}}{Z_{l}+Z_{s}}=\frac{\cos\left(\theta \right)V\rho_{l}C_{l}\rho_{s}C_{s}}{\rho_{l}C_{l}+\rho_{s}C_{s}}$$

Since there is no coating layer present, there is no change in properties across an interface, and hence the stress in the substrate ($\sigma_{0,s}$) is equal to the impact pressure ($\sigma_{1,s}$).

The Springer model computes the lifetime based on a fatigue analysis. Firstly, the fatigue knee (*b*) and the so called erosion strength ($\sigma_{e}$) are determined according to Equations (2) and (3). These equations use the fatigue knee ($b_{2}$), ultimate tensile strength ($\sigma_{U}$), endurance limit ($\sigma_{I}$) and Poisson ratio (ν) of the impacted material.

(2) $$b_{s}=\frac{b_{2,s}}{\log_{10}\left(\frac{\sigma_{U,s}}{\sigma_{I,s}}\right)}$$

(3) $$\sigma_{e,s}=\frac{4\left(b_{s}-1\right)\sigma_{U,s}}{\left(1-2\nu_{s}\right)\left(1-{\left(\frac{\sigma_{I,s}}{\sigma_{U,s}}\right)}^{b_{s}-1}\right)}$$

Springer fit the ratio of erosion strength:stress to experimental rain erosion test results using a power law according to Equation (4). The constant $a_{1}$ was initially found to be $3.7\times 10^{-4}$ [12] but later rectified to a value of $7.1\times 10^{-6}$ [67] or $7\times 10^{-6}$ [10], of which the latter expression has more commonly been used in the literature and will be used for the remainder of this work as well. The constant $a_{2}$ was found to be 5.7 in all three references. Recently, both constants were shown to be dependent on the coating material used, especially for (viscoelastic) polymeric materials [41].

(4) $$N_{i}^{*}=a_{1}{\left(\frac{\sigma_{e,s}}{\sigma_{0,s}}\right)}^{a_{2}}$$

Equation (4) describes the lifetime for repeated impacts at a single location ($N_{i}^{*}$ [-]); a more representative scenario would be a distributed rain field. In order to include it this in the model, Springer related the lifetime to the number of impacts in a certain area ($N_{i}$ [1/m^2^]) by Equation (5). It should be noted that the area is determined by the droplet diameter (*d*) and not by the size of the stress field or the damage.

(5) $$N_{i}=N_{i}^{*}\frac{4}{\pi d^{2}}$$

Substituting Equation (4) and the constants ($a_{1}=7\times 10^{-6}$ and $a_{2}=5.7$) into Equation (5) yields Equation (6), which is generally used in the literature to predict the lifetime performance of a coated substrate based on the Springer model.

(6) $$N_{i}=\frac{8.9\times 10^{-6}}{d^{2}}{\left(\frac{\sigma_{e,s}}{\sigma_{0,s}}\right)}^{5.7}$$

### 2.2. Impact on a Coated Substrate

Using a more advanced form of the Springer model, it is possible to consider a protective coating system with finite thickness, taking 1D wave propagation in this coating layer into account. In order to simplify the equations, the impedance parameters (ψ) for the solid–coating interface and liquid–coating interface are introduced by Equation (7), where an additional subscript *c* is introduced for the coating.

(7) $$\psi_{sc}=\frac{Z_{s}-Z_{c}}{Z_{s}+Z_{c}},\;\psi_{lc}=\frac{Z_{l}-Z_{c}}{Z_{l}+Z_{c}}$$

The coating thickness parameter (γ) is determined according to Equation (8). γ is the ratio between the maximum number of reflections during the impact time in a coating with thickness *h* ($k_{L}$) and the maximum number of reflections in the coating based on the acoustic properties ($k_{e}$).

(8) $$\gamma =\frac{k_{L}}{k_{e}}=\frac{\left[\frac{C_{c}}{C_{l}}\frac{d}{h}\right]}{\left[\frac{1}{\left(1-\psi_{sc}\psi_{lc}\right)}\right]}=\frac{2C_{c}Z_{c}\left(Z_{l}+Z_{s}\right)d}{C_{l}\left(Z_{c}+Z_{l}\right)\left(Z_{c}+Z_{s}\right)h}$$

Based on this ratio, an average number of impacts (*k*) is introduced according to Equation (9). It can be seen that for thick coatings, the value of γ, and hence that of *k*, reaches 0, indicating that there are no reflections in the coating. For thin coatings, γ reaches toward infinity, indicating that *k* converges to the maximum number of reflections based on the acoustic properties ($k_{e}$). The space in between is spanned by the exponential function based on γ.

(9) $$k=k_{e}\left(1-e^{-\gamma }\right)=\frac{1-e^{-\gamma }}{1-\psi_{lc}\psi_{sc}}$$

With the average number of reflections known, the stress at the liquid–coating interface ($\sigma_{0}$) and the stress at the solid–coating interface ($\sigma_{h}$), including the effect of the reflections, can be calculated according to Equations (10) and (11). The contact pressure $\sigma_{1,c}$ is Equation (1) using the coating properties instead of the substrate properties.

(10) $$\sigma_{0,c}=\sigma_{1,c}\frac{1+\psi_{sc}}{1-\psi_{sc}\psi_{lc}}\left[1-\psi_{sc}\frac{1+\psi_{lc}}{1+\psi_{sc}}\frac{1-e^{-\gamma }}{\gamma }\right]$$

(11) $$\sigma_{h,c}=\sigma_{1,c}\frac{1+\psi_{sc}}{1-\psi_{sc}\psi_{lc}}\left[1-\psi_{sc}\psi_{lc}\frac{1-e^{-\gamma }}{\gamma }\right]$$

In order to determine the lifetime of the coated substrate, the fatigue properties still need to be considered. Springer accounted for the number of reflections in calculating the erosion strength according to Equation (12), where $b_{c}$ is equal to $b_{s}$ in Equation (2) using the properties of the coating instead of the substrate.

(12) $$\sigma_{e,c}=\frac{4\left(b_{c}-1\right)\sigma_{u,c}}{\left(1-2\nu_{c}\right)\left(1-{\left(\frac{\sigma_{I,c}}{\sigma_{U,c}}\right)}^{b_{c}-1}\right)\left(2k\right|\psi_{sc}\left|+1\right)}$$

Using Equations (10) and (12) in Equations (4) and (6) gives the lifetimes for coated substrates. Depending on the properties of the coating and the substrate, the lifetime can be elongated or shortened with respect to the uncoated substrate’s lifetime.

Regarding the modeling framework proposed in Figure 2, Equation (1) can be considered as the contact pressure model, Equations (10) and (11) as the coating stress model and Equations (2), (3) and (12) as the fatigue strength model. The lifetimes are then calculated by Equations (4) and (5).

MATLAB code for solving the lifetime of a coated substrate using the Springer model is provided in Appendix A. Note that this model solves the problem for an uncoated substrate when the coating and substrate properties are considered equal.

## 3. Results

Based on the Springer model described in Section 2, the lifetimes for an arbitrary set of materials were studied and the sensitivity of the model to its input parameters was investigated.

### 3.1. Lifetime Results

**Figure 3:**
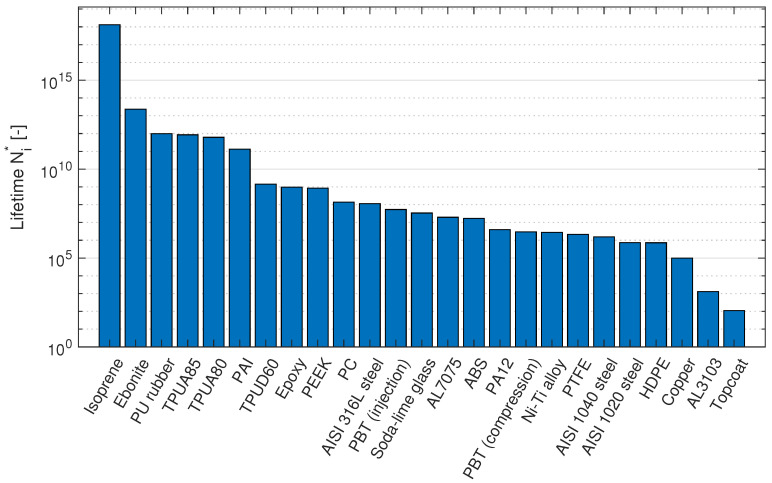
Coating lifetimes according to the Springer model.

In order to compute the lifetimes according to the Springer model, an impact velocity (*V*) of 100 ms^−1^, a droplet diameter (*d*) of 2 mm and a coating thickness (*h*) of 750 μm were considered. Table 2 shows the input material parameters obtained from the literature and GRANTA edupack [68], and the corresponding Springer lifetimes. It should be noted that by using GRANTA edupack, the *b* value is assumed to be 7 which is not necessarily an accurate representation. Fatigue tests for the materials should be performed to obtain more representative values for *b*. Figure 3 shows the modeled lifetimes. An interesting observation is that the different material classes do not show a preferred order, with the exception of elastomers, which in general show higher lifetimes than other material classes. It can also be seen that slight changes in material parameters can cause significant changes in lifetime, as seen from the two PBT variants and from the difference between TPUD60 and PA12. This last observation raises the question of how the Springer model responds to variations in its input parameters, and therefore, a sensitivity study was performed.

### 3.2. Sensitivity of the Springer Model to Its Input Parameters

In order to perform a local sensitivity study on the input parameters of the Springer model, TPUA80 and AISI 316L steel were chosen from Table 2 as reference materials, since their properties are very different. The input parameters that can be changed are mainly the coating parameters. It was therefore decided to set the liquid and substrate parameters constant and only make the coating parameters variable. Since the coating thickness can also be changed, this was included in the analysis. The sensitivity study considered the reference parameters and found the effects of variations in input parameters on the lifetimes of the coated substrates. Each of the parameters was changed in the range of 70% to 130% of its reference value.

**Figure 4:**
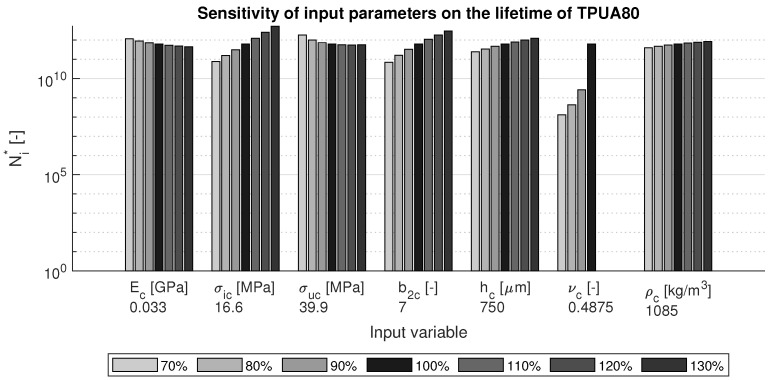
Sensitivity of variations in the input parameters on the lifetime of TPUA80.

The sensitivity study for the TPUA80 reference material is given in Figure 4. It can be seen that the largest influence on the lifetime is caused by the Poisson ratio: a lower value results in a significantly lower lifetime. The strength parameters have the second largest contribution: higher values for the fatigue endurance limit and lower values for ultimate tensile strength result in a longer lifetime.

**Figure 5:**
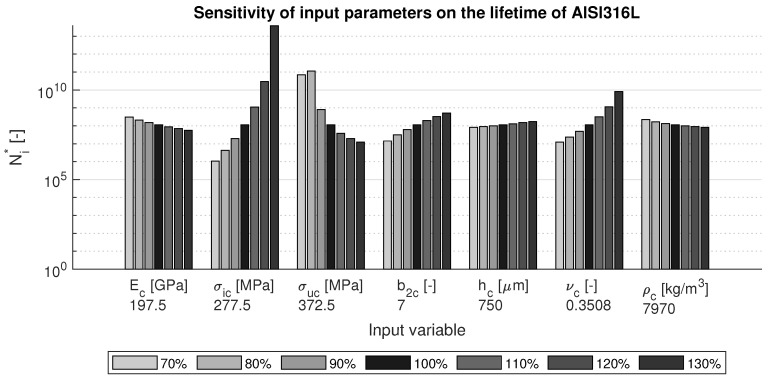
Sensitivity of variations in the input parameters on the lifetime of AISI 316L steel.

The results for AISI 316L steel are shown in Figure 5. Even though this material has very different properties, it is again seen that the Poisson ratio has a large influence on the lifetime: a higher value yields a long lifetimes. This material also shows a large dependence on the strength values: similar values for ultimate tensile strength and fatigue endurance limit are desired. Coating thickness and elastic parameters play less important roles in the sensitivity, although it should be noted that since this figure is on logarithmic scale, these parameters also change the coating lifetimes with a factor of about 10.

**Figure 6:**
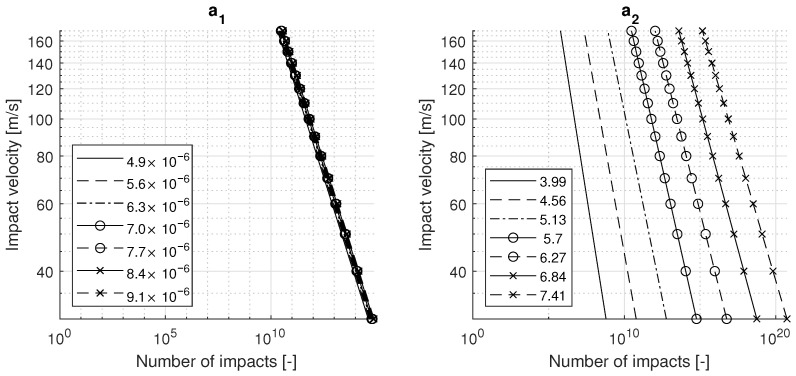
VN curves for variations in $a_{1}$ (**left**) and $a_{2}$ (**right**) for TPUA80.

In order to study the effects of the constants $a_{1}$ and $a_{2}$, the VN curve is required. This curve plots the number of impacts against the impact velocity and is generally used to display fatigue life. The VN curves for variations in the constants $a_{1}$ and $a_{2}$ are given in Figure 6. It can be seen that the effect of $a_{1}$ on the coating life is very limited. The influence of $a_{2}$ is more prominent: the graph shows significant changes in coating lifetime and the slope of the fatigue curve. By changing the ratio of $\sigma_{e,c}$ to $\sigma_{0,c}$, the curve can be shifted along the *x*-axis. This is a common approach for fitting the Springer model to rain erosion test data.

**Figure 7:**
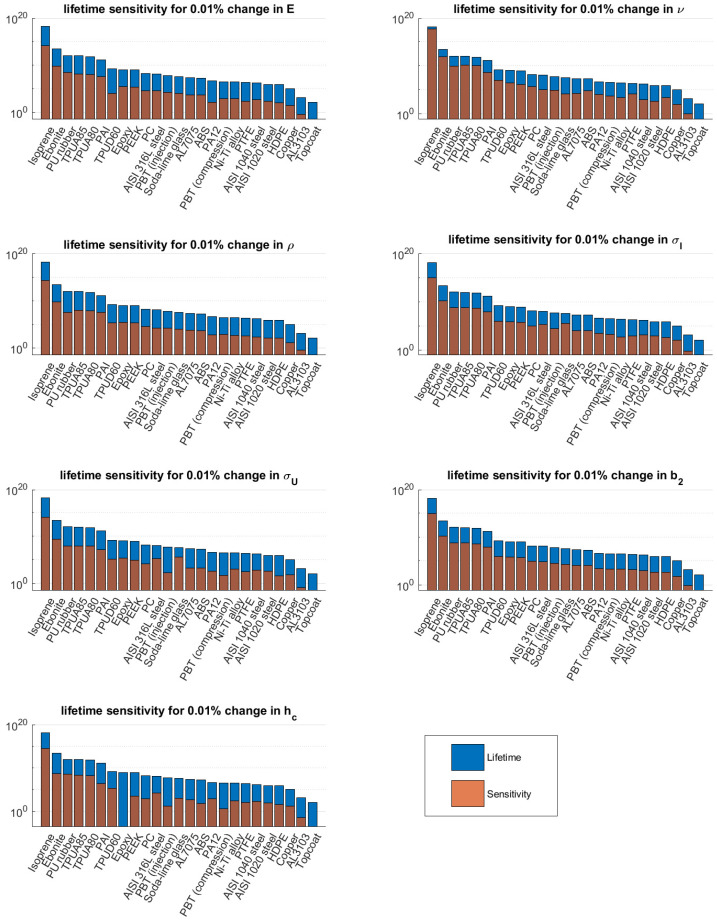
Global sensitivity analysis for the materials considered in Table 2.

The average sensitivity for the materials defined in Table 2 was determined in order to perform a global sensitivity analysis. The results can be found in Figure 7. This figure shows the predicted lifetime and the sensitivity, i.e., the change in lifetime for a variation of 0.01% in an input parameter. It can be seen that a change in Poisson ratio has a large effect on the predicted lifetimes for elastomeric materials, e.g., isoprene, ebonite and PU rubber. Moreover, it can be seen that for some materials, e.g., AISI 316L steel and soda lime-glass, the strength parameters play an important role in lifetime sensitivity. Looking at the material parameters of these materials, it can be observed that the values of $\sigma_{U,c}$ and $\sigma_{I,c}$ are close to each other or the Poisson ratio is close to 0.5.

In addition to material list in Table 2, a random set of input parameters (N = 100.000) was determined and a sensitivity analysis was performed based on variations of 0.01% in input parameters. The distribution of lifetimes and sensitivities for each of the 100.000 points are shown in Appendix B. The mean and median values for each of the input parameters were determined and are shown in Table 3. From the table, it can be observed that the highest sensitivity in both cases is caused by the Poisson ratio, endurance limit and material fatigue knee value. This observation is strengthened by the local sensitivity analyses shown in Figure 4 and Figure 5. Another interesting observation is that the set of materials defined in Table 2 is a good representation of the general average in terms of sensitivity, as seen in Table 3. The lifetimes themselves are different because in a random selection, unrealistic materials with low elasticity and high strength do exist.

Based on the random material input data, the material properties for the material with the highest lifetime were observed, and it was found that the values for $\sigma_{U,c}$ and $\sigma_{I,c}$ were practically equal. By defining a limit in the possible input values where $\sigma_{I,c}\leq 0.8\;\sigma_{U,c}$, the highest simulated lifetime reduced significantly and consisted of a material with a Poisson ratio of 0.4999, which was at the defined upper bound for the input data. This analysis indicated that materials with similar strength values and high Poisson ratios have long predicted lifetimes according to the Springer model.

## 4. Discussion

Based on the lifetimes predicted by the Springer model and the sensitivity studies, it can be concluded that according to the Springer model, an optimized solution consists of a coating material with a Poisson ratio close to 0.5, indicating incompressibility, or a coating material with a similar ultimate tensile strength and fatigue endurance limit. This preference is due to the fact that Springer uses these values to calculate $\sigma_{e}$. In order for $N_{i}^{*}$ or $N_{i}$ to be large, the erosion strength has to be large. The denominator in Equation (12) becomes small for ν→0.5 and the nominator goes to infinity for $\frac{\sigma_{U,c}}{\sigma_{I,c}}\to 1$, resulting in a high erosion strength and hence a long lifetime. The physical meanings of these relations are unknown, and therefore, the Springer model should be used with caution.

Moreover, it has been shown by sensitivity studies of the constants $a_{1}$ and $a_{2}$ that the coating lifetime is highly sensitive to changes in $a_{2}$. Since these parameters are determined by an empirical fit of rain erosion test results, their accuracies and physical meanings are unknown. A high sensitivity of the model to such parameters is undesired, and this should be considered when using the Springer model. Additionally, a coating lifetime prediction model should be able to predict the rain erosion test results based on available material models; it should not require empirical fitting to the desired results.

Since the Springer model was developed without the availability of computational methods, it was based on analytical descriptions. This allows for fast and easy computation, but also involves simplifications. The first simplification was made in determining the contact pressure. This was based on the (modified) waterhammer pressure, which consists of a scalar value which scales linearly with impact velocity. This parameter, however, is independent of time, droplet size and distance to the center of impact, which have all been shown to play important roles. In order to accurately predict contact pressure, a sophisticated dynamic contact pressure model that governs the physical droplet impact phenomena is required.

It is noteworthy that the linear relation between the impact velocity and stress modeled by the waterhammer pressure is also used for interpretation of rain erosion test results by using the VN curve instead of the true SN curve which is based on the stress. The relation between stress and impact velocity is not necessarily linear, which could lead to misinterpretations of the results or at least difficulties in relating RET results to fatigue material data. Moreover, the Springer model uses the droplet diameter to convert $N_{i}^{*}$ into $N_{i}$ (as shown in Equation (5)), whereas in reality the interactions of multiple impact locations are dependent on the size of the stress field and the damaged area.

The second part of the Springer model assumes one-dimensional wave propagation in the substrate. This allows for a simplified analytical analysis of the stress in the impacted material. In reality, however, the stress field is three dimensional and its different components play distinct roles. The magnitude and propagation of these distinct waves in all three dimensions depend significantly on the material’s parameters. In order to accurately predict LEP performance, a detailed 3D dynamic stress model which considers the dynamic impact pressure is required.

The third part of the Springer model concerns the fatigue strength analysis. For this purpose, the Springer model utilizes the Wöhler fatigue data at ambient low-strain rate conditions. The strain rates due to liquid droplet impact, however, are significantly higher. Depending on the materials that are used, the high-strain rate properties can differ significantly from the low-strain rate properties. It is therefore required to consider high-strain rate fatigue properties. These can be obtained by time-temperature superposition of dynamic mechanical thermal analysis (DMTA) test results, or by high rate testing with, for example, a split Hopkinson pressure bar. Another method would be to compare the results with RET data, but this is a more empirical approach. To develop a physics based model that predicts the RET performance, high-strain rate properties of the coating materials need to be characterized experimentally.

## 5. Conclusions

This paper reviewed the most used model to predict the lifetime of leading edge protection systems for wind turbine blades, the Springer model. We showed that the philosophy behind the Springer model is very powerful and could lead to sophisticated coupling between liquid droplet impact pressure, stress wave propagation in elastic solids and high-rate fatigue behavior. The Springer model was separated into three distinct parts:

1. Contact pressure model;
2. Coating stress model;
3. Fatigue strength model.

These models are solved consecutively to obtain an estimation of the coating lifetime. The sensitivity of the predicted lifetimes to changes in the input parameters was calculated and analyzed.

- Traditionally, the Springer model uses the static Waterhammer pressure as a basis for the contact pressure model, a one-dimensional coating reflection model for the coating stress and low-rate fatigue properties for the fatigue strength. The lifetime is fitted using empirical relations with erosion experiments. Recently, more advanced coating materials are being developed for which the traditional Springer model is no longer able to accurately predict LEP performance due to a lack of physical representation by these assumptions.
- The current work analyzed the sensitivity of the Springer model with respect to its input parameters and showed that it is most sensitive to the Poisson ratio, the strength values and the empirically fitted constants.. It was shown that a Poisson ratio of 0.5 and similar values of the ultimate tensile strength and the fatigue limit lead to high values for the erosion strength, and therefore to long lifetime predictions. The physical meanings of these relations, however, are still not explored, and it is undesirable for the model to be sensitive to input parameters that are close to these bounds.
- In order to accurately model LEP lifetimes according to the framework presented in Figure 2, proper descriptions of the three subcomponents of the model are required. The traditional Springer model can give a quick estimation of LEP performance; however, the accuracy of its predictions need to be improved. Utilizing numerical computational models in order to obtain representative physical data on both contact pressure and stress in the coating material, in addition to using high-rate fatigue properties, would yield a more accurate estimation of LEP performance and more insights into the erosion mechanisms and should therefore be the focus of future research.

## Figures and Tables

**Table 1:** Used quantities and corresponding units in the Springer model.

Quantity	Unit	Liquid	Coating	Substrate
Impact velocity, *V*	(m/s)	-	-	-
Droplet diameter, *d*	(m)	-	-	-
Impact angle, θ	(${}^{\circ }$)	-	-	-
Coating thickness, *h*	(m)	-	-	-
Density, ρ	(kg/m${}^{3}$)	$\rho_{l}$	$\rho_{c}$	$\rho_{s}$
Acoustic velocity, *C*	(m/s)	$C_{l}$	$C_{c}$	$C_{s}$
Springer fatigue knee, *b*	(-)	-	$b_{c}$	$b_{s}$
Material fatigue knee, $b_{2}$	(-)	-	$b_{2,c}$	$b_{2,s}$
Ultimate tensile strength, $\sigma_{U}$	(Pa)	-	$\sigma_{U,c}$	$\sigma_{U,s}$
Endurance limit, $\sigma_{I}$	(Pa)	-	$\sigma_{I,c}$	$\sigma_{I,s}$
Poisson ratio, ν	(-)	-	$\nu_{c}$	$\nu_{s}$

**Table 2:** Material parameters and lifetimes according to the Springer model for arbitrary materials.

Material	$E_{c}$ (GPa)	$\nu_{c}$ (-)	$\rho_{c}$ (kg/m^3^)	$\sigma_{U,c}$ (MPa)	$\sigma_{I,c}$ (MPa)	$b_{2,c}$ (-)	$N_{I}^{*}$ (-)
Isoprene	0.00215	0.4995	950	24.4	9.75	7	1.32 × 10${}^{18}$
Ebonite	1.5	0.4945	1180	70	28	7	2.32 × 10${}^{13}$
PU rubber	0.01625	0.48	1200	45.5	18.2	7	9.91 × 10${}^{11}$
TPUA85	0.03415	0.4875	1195	43.7	17.5	7	8.50 × 10${}^{11}$
TPUA80	0.033	0.4875	1085	39.9	16.6	7	6.15 × 10${}^{11}$
PAI	4.9	0.45	1425	192	77	7	1.31 × 10${}^{11}$
TPUD60	0.25	0.4575	1100	55.1	22.05	7	1.44 × 10${}^{9}$
Epoxy	2.41	0.399	1255	67.3	32.5	7	9.56 × 10${}^{8}$
PEEK	3.855	0.4	1310	107	43	7	8.43 × 10${}^{8}$
PC	2.305	0.4	1160	63.1	25.45	7	1.41 × 10${}^{8}$
AISI 316L steel	197.5	0.35075	7970	372.5	277.5	7	1.14 × 10${}^{8}$
PBT (injection) [17]	2.545	0.4	1370	65.8	22.4	7	5.34 × 10${}^{7}$
Soda-lime glass	69.95	0.215	2465	32.6	30.95	7	3.40 × 10${}^{7}$
AL7075	72.5	0.33	2800	555	160	7	1.97 × 10${}^{7}$
ABS	2.45	0.408	1050	40	16	7	1.68 × 10${}^{7}$
PA12	0.385	0.414	1035	40	16	7	3.94 × 10${}^{6}$
PBT (compression) [17]	3.16	0.4	1370	45.4	14.9	7	2.84 × 10${}^{6}$
Ni-Ti alloy	34.5	0.33	6475	1397.5	35.15	7	2.75 × 10${}^{6}$
PTFE	0.476	0.45	2170	27.6	6.375	7	2.15 × 10${}^{6}$
AISI 1040 steel	212	0.29	7850	525	272.5	7	1.56 × 10${}^{6}$
AISI 1020 steel	210	0.29	7850	395	223.5	7	7.28 × 10${}^{5}$
HDPE	1.08	0.4185	958.5	28.5	10.62	7	7.22 × 10${}^{5}$
Copper	125	0.345	8945	152.5	94	7	9.99 × 10${}^{4}$
AL3103	71.25	0.33	2730	105	28.7	7	1.28 × 10${}^{3}$
Topcoat [41]	3.81	0.295	1690	13	6.316	5.2	1.10 × 10${}^{2}$

**Table 3:** Average sensitivity for the global sensitivity analysis.

Material Set in Table 2	*E*	ν	ρ	$\sigma_{I}$	$\sigma_{U}$	$b_{2}$	$h_{c}$
mean sensitivity (exponent)	4.4554	5.6416	4.4698	5.0851	4.3542	4.9944	3.7044
std sensitivity (exponent)	3.3636	4.0112	3.3569	3.4238	3.3801	3.4167	3.8306
median sensitivity (exponent)	3.9261	4.7511	3.9677	4.4808	3.1787	4.2892	2.7423
median lifetime (exponent)	7.532	7.532	7.532	7.532	7.532	7.532	7.532
median sensitivity	8435.2	56,373	9282.8	30,253	1509	19,463	569.51
median lifetime	3.40 × 10${}^{7}$	3.40 × 10${}^{7}$	3.40 × 10${}^{7}$	3.40 × 10${}^{7}$	3.40 × 10${}^{7}$	3.40 × 10${}^{7}$	3.40 × 10${}^{7}$
**100.000 Random Points**	E	ν	ρ	$\sigma_{I}$	$\sigma_{U}$	$b_{2}$	$h_{c}$
mean sensitivity (exponent)	4.6557	5.3222	4.4546	5.279	4.9348	5.0278	3.9066
std sensitivity (exponent)	4.0409	4.2452	4.0705	4.4837	4.5973	4.0249	4.049
median sensitivity (exponent)	4.2722	4.9122	4.0802	4.8041	4.335	4.6395	3.5339
median lifetime (exponent)	7.8597	7.8597	7.8597	7.8597	7.8597	7.8597	7.8597
median sensitivity	18,716	81,696	12,029	63,700	21,627	43,600	3419.2
median lifetime	7.24 × 10${}^{7}$	7.24 × 10${}^{7}$	7.24 × 10${}^{7}$	7.24 × 10${}^{7}$	7.24 × 10${}^{7}$	7.24 × 10${}^{7}$	7.24 × 10${}^{7}$

**Figure A1:**
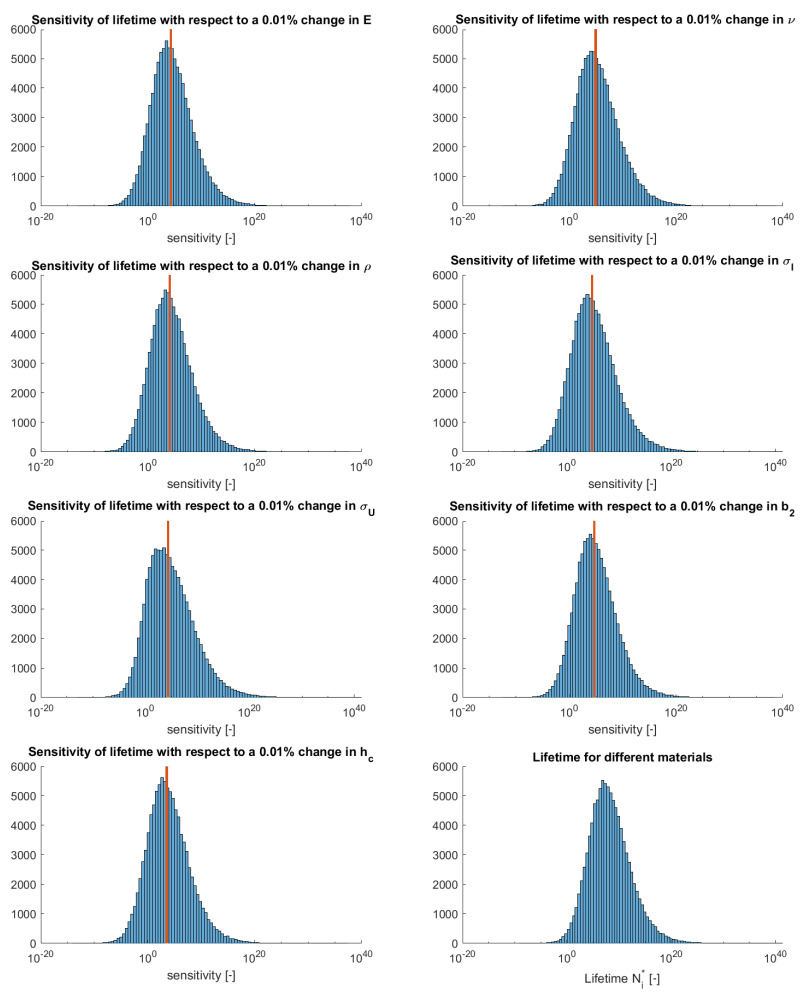
Distributions of sensitivities and lifetimes for the global sensitivity analysis.

## Data Availability

Not applicable.

## Appendix B. Global Sensitivity Analysis for 100.000 Random Input Points

The global sensitivity analysis performed considered 100,000 input points randomly distributed inside the 7-parameter space bound by the values given in Table A1. The resulting distributions of lifetimes and sensitivities arer given in Figure A1. The red line depicts the median value for the corresponding parameter.

**Table A1:** Bounds used for each of the input parameters for the global sensitivity study.

	*E*	ν	ρ	$\sigma_{U}$	$\sigma_{I}$	$b_{2}$	$h_{c}$
Lower bound	1.00 × 10${}^{6}$	0.2	800	5.00 × 10${}^{5}$	5.00 × 10${}^{5}$	5	1.00 × 10${}^{-4}$
Upper bound	2.00 × 10${}^{11}$	0.4999	7500	5.00 × 10${}^{8}$	$\sigma_{U}$	9	2.00× 10${}^{-3}$

## References

[1] Shohag M.A.S., Hammel E.C., Olawale D.O., Okoli O.I. (2017). Damage mitigation techniques in wind turbine blades: A review. Wind. Eng..

[2] Katsaprakakis D.A., Papadakis N., Ntintakis I. (2021). A comprehensive analysis of wind turbine blade damage. Energies.

[3] Mishnaevsky L., Hasager C.B., Bak C., Tilg A.M.M., Bech J.I., Doagou Rad S., Fæster S. (2021). Leading edge erosion of wind turbine blades: Understanding, prevention and protection. Renew. Energy.

[4] Castorrini A., Corsini A., Rispoli F., Venturini P., Takizawa K., Tezduyar T.E. (2019). Computational analysis of performance deterioration of a wind turbine blade strip subjected to environmental erosion. Comput. Mech..

[5] Papi F., Ferrara G., Bianchini A. (2020). Practical Considerations on Wind Turbine Blade Leading Edge Erosion Modelling and its Impact on Performance and Loads. J. Phys. Conf. Ser..

[6] Papi F., Cappugi L., Perez-Becker S., Bianchini A. (2020). Numerical modeling of the effects of leading-edge erosion and trailing-edge damage on wind turbine loads and performance. J. Eng. Gas Turbines Power.

[7] Mishnaevsky L. (2019). Repair of wind turbine blades: Review of methods and related computational mechanics problems. Renew. Energy.

[8] Dashtkar A., Hadavinia H., Sahinkaya M.N., Williams N.A., Vahid S., Ismail F., Turner M. (2019). Rain erosion-resistant coatings for wind turbine blades: A review. Polym. Polym. Compos..

[9] Major D., Palacios J., Maughmer M., Schmitz S. (2021). Aerodynamics of leading-edge protection tapes for wind turbine blades. Wind. Eng..

[10] Springer G.S. (1976). Erosion by Liquid Impact.

[11] Verma A.S., Castro S.G.P., Jiang Z., Teuwen J.J.E. (2020). Numerical investigation of rain droplet impact on offshore wind turbine blades under different rainfall conditions: A parametric study. Compos. Struct..

[12] Springer G.S., Baxi C.B. (1972). A Model for Rain Erosion of Homogeneous Materials (Water Impingement Model for Raindrop Erosion of Homogeneous Materials) [Technical Report, June 1971–May 1972]. https://deepblue.lib.umich.edu/handle/2027.42/7754.

[13] Zhou Q., Li N., Chen X., Xu T., Hui S., Zhang D. (2008). Liquid drop impact on solid surface with application to water drop erosion on turbine blades, Part II: Axisymmetric solution and erosion analysis. Int. J. Mech. Sci..

[14] Zhou Q., Li N., Chen X., Xu T., Hui S., Zhang D. (2009). Analysis of water drop erosion on turbine blades based on a nonlinear liquid–solid impact model. Int. J. Impact Eng..

[15] Eisenberg D., Laustsen S., Stege J. (2018). Wind turbine blade coating leading edge rain erosion model: Development and validation. Wind. Energy.

[16] Slot H.M., Gelinck E.R.M., Rentrop C., Van der Heide E. (2015). Leading edge erosion of coated wind turbine blades: Review of coating life models. Renew. Energy.

[17] Slot H.M., IJzerman R.M., le Feber M., Nord-Varhaug K., van der Heide E. (2018). Rain erosion resistance of injection moulded and compression moulded polybutylene terephthalate PBT. Wear.

[18] Slot H., Matthews D., Schipper D., van der Heide E. (2021). Fatigue-based model for the droplet impingement erosion incubation period of metallic surfaces. Fatigue Fract. Eng. Mater. Struct..

[19] Verma A.S., Jiang Z., Caboni M., Verhoef H., van der Mijle Meijer H., Castro S.G.P., Teuwen J.J.E. (2021). A probabilistic rainfall model to estimate the leading-edge lifetime of wind turbine blade coating system. Renew. Energy.

[20] Hasager C., Vejen F., Bech J.I., Skrzypiński W.R., Tilg A.M., Nielsen M. (2020). Assessment of the rain and wind climate with focus on wind turbine blade leading edge erosion rate and expected lifetime in Danish Seas. Renew. Energy.

[21] Hasager C.B., Vejen F., Skrzypiński W.R., Tilg A.M. (2021). Rain erosion load and its effect on leading-edge lifetime and potential of erosion-safe mode at wind turbines in the north sea and baltic sea. Energies.

[22] Herring R., Dyer K., Howkins P., Ward C. (2020). Characterisation of the offshore precipitation environment to help combat leading edge erosion of wind turbine blades. Wind. Energy Sci..

[23] Pugh K., Stack M.M. (2021). Rain Erosion Maps for Wind Turbines Based on Geographical Locations: A Case Study in Ireland and Britain. J. Bio-Tribo-Corros..

[24] Nash J.W., Zekos I., Stack M.M. (2021). Mapping of meteorological observations over the island of ireland to enhance the understanding and prediction of rain erosion in wind turbine blades. Energies.

[25] Verma A.S., Jiang Z., Ren Z., Caboni M., Verhoef H., van der Mijle-Meijer H., Castro S.G., Teuwen J.J. (2021). A probabilistic long-term framework for site-specific erosion analysis of wind turbine blades: A case study of 31 Dutch sites. Wind Energy.

[26] Letson F., Barthelmie R.R., Pryor S.S. (2020). Radar-derived precipitation climatology for wind turbine blade leading edge erosion. Wind. Energy Sci..

[27] Skrzypiński W.R., Bech J.I., Hasager C.B., Tilg A.M., Bak C., Vejen F. (2020). Optimization of the erosion-safe operation of the IEA Wind 15 MW Reference Wind Turbine. J. Phys. Conf. Ser..

[28] Cappugi L., Castorrini A., Bonfiglioli A., Minisci E., Campobasso M.S. (2021). Machine learning-enabled prediction of wind turbine energy yield losses due to general blade leading edge erosion. Energy Convers. Manag..

[29] Castorrini A., Venturini P., Corsini A., Rispoli F. (2021). Machine learnt prediction method for rain erosion damage on wind turbine blades. Wind Energy.

[30] Nasiri S., Khosravani M.R. (2021). Machine learning in predicting mechanical behavior of additively manufactured parts. J. Mater. Res. Technol..

[31] Law H., Koutsos V. (2020). Leading edge erosion of wind turbines: Effect of solid airborne particles and rain on operational wind farms. Wind Energy.

[32] Prieto R., Karlsson T. (2021). A model to estimate the effect of variables causing erosion in wind turbine blades. Wind Energy.

[33] Bartolomé L., Teuwen J. (2019). Prospective challenges in the experimentation of the rain erosion on the leading edge of wind turbine blades. Wind Energy.

[34] Mackie C., Nash D., Boyce D., Wright M., Dyer K. Characterisation of a Whirling Arm Erosion Test Rig. Proceedings of the 2018 Asian Conference on Energy, Power and Transportation Electrification, ACEPT 2018.

[35] DNVGL (2018). RP-0171, Testing of Rotor Blade Erosion Protection Systems; Technical Report; DNVGL. https://www.dnv.com/energy/standards-guidelines/dnv-rp-0171-testing-of-rotor-blade-erosion-protection-systems.html.

[36] Fraisse A., Bech J.I., Borum K.K., Fedorov V., Frost-Jensen Johansen N., McGugan M., Mishnaevsky L., Kusano Y. (2018). Impact fatigue damage of coated glass fibre reinforced polymer laminate. Renew. Energy.

[37] Johansen N.F.J., Mishnaevsky L., Dashtkar A., Williams N.A., Fæster S., Silvello A., Cano I.G., Hadavinia H. (2021). Nanoengineered graphene-reinforced coating for leading edge protection of wind turbine blades. Coatings.

[38] Pugh K., Nash J.W., Reaburn G., Stack M.M. (2021). On analytical tools for assessing the raindrop erosion of wind turbine blades. Renew. Sustain. Energy Rev..

[39] Domenech L., Renau J., Šakalyte A., Sánchez F. (2020). Top coating anti-erosion performance analysis in wind turbine blades depending on relative acoustic impedance. Part 1: Modelling approach. Coatings.

[40] Domenech L., García-Peñas V., Šakalytė A., Puthukara Francis D., Skoglund E., Sánchez F. (2020). Top Coating Anti-Erosion Performance Analysis in Wind Turbine Blades Depending on Relative Acoustic Impedance. Part 2: Material Characterization and Rain Erosion Testing Evaluation. Coatings.

[41] Herring R., Domenech L., Renau J., Šakalytė A., Ward C., Dyer K., Sánchez F. (2021). Assessment of a wind turbine blade erosion lifetime prediction model with industrial protection materials and testing methods. Coatings.

[42] Cortés E., Sánchez F., O’Carroll A., Madramany B., Hardiman M., Young T.M. (2017). On the material characterisation of wind turbine blade coatings: The effect of interphase coating-laminate adhesion on rain erosion performance. Materials.

[43] Herring R., Dyer K., Martin F., Ward C. (2019). The increasing importance of leading edge erosion and a review of existing protection solutions. Renew. Sustain. Energy Rev..

[44] Hoksbergen T.H., Baran I., Akkerman R. (2020). Rain droplet erosion behavior of a thermoplastic based leading edge protection system for wind turbine blades. IOP Conf. Ser. Mater. Sci. Eng..

[45] Zanjani J.S.M., Baran I., Akkerman R. (2020). Combatting rain erosion of offshore wind turbine blades by co-bonded thermoplastic-thermoset hybrid composites. IOP Conf. Ser. Mater. Sci. Eng..

[46] Zanjani J.S.M., Baran I., Akkerman R. (2020). Characterization of interdiffusion mechanisms during co-bonding of unsaturated polyester resin to thermoplastics with different thermodynamic affinities. Polymer.

[47] Zanjani J.S.M., Baran I. (2021). Co-bonded hybrid thermoplastic-thermoset composite interphase: Process-microstructure-property correlation. Materials.

[48] Keegan M.H., Nash D.H., Stack M.M. (2012). Modelling rain drop impact of offshore wind turbine blades. Proc. ASME Turbo Expo.

[49] Keegan M.H., Nash D.H., Stack M.M. (2013). On erosion issues associated with the leading edge of wind turbine blades. J. Phys. D Appl. Phys..

[50] Doagou-Rad S., Mishnaevsky L., Bech J.I. (2020). Leading edge erosion of wind turbine blades: Multiaxial critical plane fatigue model of coating degradation under random liquid impacts. Wind. Energy.

[51] Hu W., Chen W., Wang X., Jiang Z., Wang Y., Verma A.S., Teuwen J.J. (2021). A computational framework for coating fatigue analysis of wind turbine blades due to rain erosion. Renew. Energy.

[52] Amirzadeh B., Louhghalam A., Raessi M., Tootkaboni M. (2017). A computational framework for the analysis of rain-induced erosion in wind turbine blades, part II: Drop impact-induced stresses and blade coating fatigue life. J. Wind. Eng. Ind. Aerodyn..

[53] Amirzadeh B., Louhghalam A., Raessi M., Tootkaboni M. (2017). A computational framework for the analysis of rain-induced erosion in wind turbine blades, part I: Stochastic rain texture model and drop impact simulations. J. Wind. Eng. Ind. Aerodyn..

[54] Hicks P.D., Purvis R. (2013). Liquid-solid impacts with compressible gas cushioning. J. Fluid Mech..

[55] Henman N.I.J., Smith F.T., Tiwari M.K. (2021). Pre-impact dynamics of a droplet impinging on a deformable surface. Phys. Fluids.

[56] Mishnaevsky L. (2019). Toolbox for optimizing anti-erosion protective coatings of wind turbine blades: Overview of mechanisms and technical solutions. Wind Energy.

[57] Mishnaevsky L., Fæster S., Doagou Rad S. (2020). Mechanisms and computational analysis of leading edge erosion of wind turbine blades. IOP Conf. Ser. Mater. Sci. Eng..

[58] Mishnaevsky L., Sütterlin J. (2019). Micromechanical model of surface erosion of polyurethane coatings on wind turbine blades. Polym. Degrad. Stab..

[59] Fæster S., Johansen N.F.J., Mishnaevsky L., Kusano Y., Bech J.I., Madsen M.B. (2021). Rain erosion of wind turbine blades and the effect of air bubbles in the coatings. Wind Energy.

[60] Mishnaevsky L., Fæster S., Mikkelsen L.P., Kusano Y., Bech J.I. (2020). Micromechanisms of leading edge erosion of wind turbine blades: X-ray tomography analysis and computational studies. Wind Energy.

[61] Doagou-Rad S., Mishnaevsky L. (2020). Rain erosion of wind turbine blades: Computational analysis of parameters controlling the surface degradation. Meccanica.

[62] Yadav A., Godara R., Bhardwaj G. (2020). A review on XIGA method for computational fracture mechanics applications. Eng. Fract. Mech..

[63] Verma A.S., Castro S.G., Jiang Z., Hu W., Teuwen J.J. (2020). Leading edge erosion of wind turbine blades: Effects of blade surface curvature on rain droplet impingement kinematics. J. Phys. Conf. Ser..

[64] Groucott S., Pugh K., Zekos I., Stack M.M. (2021). A study of raindrop impacts on a wind turbine material: Velocity and impact angle effects on erosion maps at various exposure times. Lubricants.

[65] Ibrahim M.E., Medraj M. (2020). Water droplet erosion ofwind turbine blades: Mechanics, testing, modeling and future perspectives. Materials.

[66] Chen J., Wang J., Ni A. (2019). A review on rain erosion protection of wind turbine blades. J. Coat. Technol. Res..

[67] Springer G.S., Yang C.I., Larsen P.S. (1974). Analysis of Rain Erosion of Coated Materials. J. Compos. Mater..

[68] (2020). Ansys Granta EduPack Software.

